# Rural-Urban Disparities in the Surgical Treatment of Carpal Tunnel Syndrome in the United States

**DOI:** 10.7759/cureus.65687

**Published:** 2024-07-29

**Authors:** Brandon Earp, Philip Blazar, Dafang Zhang

**Affiliations:** 1 Orthopaedic Surgery, Brigham and Women's Hospital, Boston, USA; 2 Orthopaedic Surgery, Harvard Medical School, Boston, USA

**Keywords:** rural-urban disparity, rural health, health disparity, carpal tunnel syndrome, global healthcare systems

## Abstract

Purpose: Rural populations are at risk for poorer access to health services and lower quality care, and recent policy efforts have focused on the reduction of rural-urban health inequities. The objective of this study was to identify differences in (1) patient demographic factors, (2) the utilization of confirmatory electrodiagnostic (EDS) testing, and (3) preoperative EDS severity between rural and urban populations undergoing carpal tunnel release (CTR).

Methods: We retrospectively identified 1,297 patients who underwent CTR at a tertiary referral center from July 2008 to June 2013. Exclusion criteria were acute trauma or infection, revision surgery, incomplete medical records, neoplasm excision, and the lack of rural-urban commuting area (RUCA) code for rural-urban classification. A final cohort of 1,138 patients who underwent CTR were included. The RUCA was used to classify patients by rural or urban residence. We assessed patient demographic factors including comorbidities, the utilization of confirmatory EDS testing, and preoperative EDS severity. A bivariate screen was performed for associations between rural-urban residence and our outcome variables, and variables with p <0.05 in the bivariate screen were included in a multivariable logistic regression model.

Results: Of the 1,138 patients, 55 patients (5%) resided in a rural area and 1,083 patients (95%) resided in an urban area. No difference was found in the utilization of confirmatory EDS testing between rural and urban patients undergoing CTR. The final multivariable logistic regression model showed that rural residence was independently associated with older age, lower body mass index (BMI), and EDS-negative disease.

Conclusions: Rural patients undergoing CTR are more likely to have EDS-negative disease, which calls into question the effectiveness of EDS testing as a confirmatory test in this population. There is a paucity of literature on the outcomes of CTR in a rural population, and further studies are needed to ensure rural-urban equity in care.

## Introduction

Carpal tunnel syndrome (CTS) is the most common peripheral nerve entrapment, with an estimated prevalence between 3% and 5% of the general population [1]. The diagnosis of CTS is established by clinical history and physical examination, with or without the aid of further confirmatory testing [2-4]. Carpal tunnel release (CTR) is a common, safe, and effective surgical treatment of CTS [5-7].

There has been a recent focus on the reduction of rural-urban health inequities [8-10]. Rural areas are defined broadly to describe regions with low or diffuse population densities, and 15% of the overall US population resides in rural communities [11]. Patients living in rural areas have poorer self-reported health status, higher rates of medical comorbidities such as diabetes mellitus, obesity, and hypertension, and higher rates of behavioral risk factors such as smoking [11,12]. Rural patients are at higher risk for low health literacy and poverty. However, because the rural healthcare infrastructure and workforce are limited, patients residing in rural locations often incur higher costs and more travel time to access healthcare resources. These socioeconomic factors compound rural-urban health disparities [11].

Rural-urban disparities in hand surgery have not been previously studied. We chose CTS as a model to study rural-urban disparities in hand surgery, as CTR is a common, elective, ambulatory procedure, that does not require heavy resources. It is not clear whether rural patients and urban patients who present for CTR are similar demographically and whether they present in similar stages of the disease. With this gap in knowledge, we aimed to identify differences in (1) patient demographic factors, (2) the utilization of confirmatory electrodiagnostic (EDS) testing, and (3) preoperative EDS severity between rural and urban patient populations undergoing CTR.

## Materials and methods

Study design and patient identification

With the approval of our institutional review board, we retrospectively identified all patients who underwent CTR surgery by one of four subspecialty-certified hand surgeons at a single metropolitan tertiary care referral center over a five-year time period from July 2008 to June 2013. Patients were identified by querying the billing records database using the Common Procedural Terminology (CPT) code for patients who underwent open CTR (CPT code 64721: neuroplasty and/or transposition; median nerve at the carpal tunnel). No endoscopic CTR was performed at our institution within the study period.

**Figure 1:**
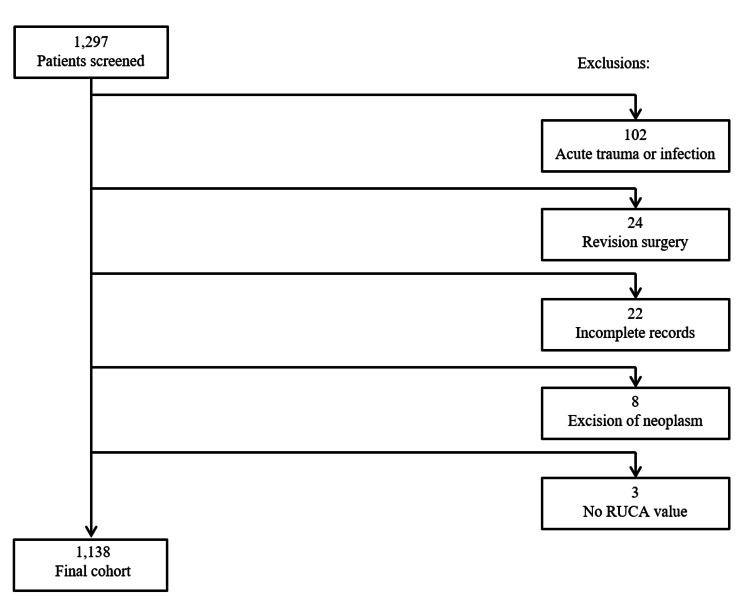
Study inclusion flow diagram. RUCA, rural-urban commuting area.

The initial query yielded 1,297 patients who underwent CTR within the study period. Patients were excluded from this study for acute trauma or infection within two weeks prior to CTR (n = 102), the CTR in question being revision surgery (n = 24), incomplete or unavailable medical records (n = 22), and excision of neoplasm (n = 8). Additionally, patients were excluded from this study if they resided in a zip code without a corresponding rural-urban commuting area (RUCA) code allowing for rural-urban classification (n = 3). A final cohort of 1,138 patients who underwent CTR were included in the study (Figure 1).

Determination of rural-urban residence

Patients were dichotomized and determined to reside in a rural or urban area using the RUCA code. The United States Department of Agriculture uses the RUCA code to classify areas based on population density, urbanization, and daily commuting. The most recently available 2010 RUCA codes, based on the 2010 decennial US census data, were used for this study [13]. The RUCA code ranges from 1 to 10; patients residing in an area with RUCA code 1 to 3 were designated urban, and patients residing in an area with RUCA code 4 or greater were designated rural, in accordance with multiple prior studies [9,10,14].

Outcome variables

The primary outcome variables were (1) patient demographic factors, (2) the utilization of confirmatory EDS testing, and (3) preoperative EDS severity. Patient demographic factors studied included age, sex, body mass index (BMI), English language speaker, and the presence of medical comorbidities. Medical comorbidities were assessed by review of the electronic medical records and included diabetes mellitus, hypertension, hypothyroidism, chronic kidney disease, cervical radiculopathy, and active tobacco use. Preoperative confirmatory EDS testing was obtained at the discretion of the treating surgeon, and EDS severity was graded as normal, mild, moderate, or severe in accordance with established practice guidelines at the discretion of the treating neurophysiologist [15,16].

Statistical analysis

Descriptive statistics for the study cohort were calculated. Parametric continuous variables were described using means and standard deviations, non-parametric continuous variables were described using medians and interquartile ranges, and categorical variables were described using percentages. Variables were analyzed using complete datasets. There was greater than 95% data completeness for all patient demographic variables. Of the 1,003 out of 1,138 patients who underwent preoperative confirmatory EDS testing, the EDS results were available for 918 patients (92%) which allowed grading of severity.

A bivariate screen was performed for associations between rural-urban residence and our outcome variables. The Student’s t-test was employed for parametric continuous variables, the Mann-Whitney U test for non-parametric continuous variables, and the Fisher's exact test for categorical variables. We included variables with p-values <0.05 in the bivariate screen in a multivariable logistic regression model to determine independent association with rural-urban residence. The standard significance criterion of α =0.05 was utilized.

## Results

Characteristics of the study cohort

The mean age of the 1,138 patients in the final cohort was 58 years, the median BMI was 30, and 69% were female. Nineteen percent of patients had diabetes mellitus, 14% had hypothyroidism, 13% used tobacco, and 9% had cervical radiculopathy. Preoperative confirmatory EDS testing was obtained in 1,003 patients (88%). Preoperative EDS severity grading was available in 918 patients and was normal in 3%, mild in 20%, moderate in 47%, and severe in 30% (Table 1).

**Table 1:** Characteristics of the study cohort (n = 1,138). ^†^Data was missing for the following explanatory variables: English speaker (n = 46), diabetes mellitus (n = 3), hypertension (n = 3), hypothyroidism (n = 3), chronic kidney disease (n = 3), and tobacco use (n = 9). Of the 1,003 patients with confirmatory EDS testing, severity was available for 918. EDS, electrodiagnostic.

Variable^†^	Total cohort (n = 1,138)	Rural cohort (n = 55)	Urban cohort (n = 1,083)	p-Value
	Mean (SD)	Mean (SD)	Mean (SD)	
Age	58.4 (14.1)	63.1 (11.6)	58.2 (14.1)	<0.05
	Median (IQR)	Median (IQR)	Median (IQR)	
Body mass index (BMI)	29.7 (25.7-34.6)	27.2 (23.6-31.5)	29.8 (25.8-34.7)	<0.05
	n (%)	n (%)	n (%)	
Female sex	783 (68.8)	34 (61.8)	749 (69.2)	0.3
English speaker	999 (91.5)	52 (98.1)	947 (91.2)	0.08
Confirmatory EDS testing	1,003 (88.2)	46 (83.6)	957 (88.4)	0.3
EDS grade normal	28 (3.1)	5 (12.5)	23 (2.6)	<0.05
EDS grade mild	187 (20.4)	3 (7.5)	184 (21.0)	<0.05
EDS grade moderate	432 (47.1)	18 (45.0)	414 (47.2)	0.9
EDS grade severe	271 (29.5)	14 (35.0)	257 (29.3)	0.5
Diabetes mellitus	213 (18.8)	6 (10.9)	207 (19.2)	0.2
Hypertension	540 (47.6)	26 (47.3)	514 (47.6)	0.9
Hypothyroidism	155 (13.7)	6 (10.9)	149 (13.8)	0.7
Chronic kidney disease	32 (2.8)	4 (7.3)	28 (2.6)	0.06
Cervical radiculopathy	99 (8.7)	2 (3.6)	97 (9.0)	0.2
Tobacco use	147 (13.0)	7 (12.7)	140 (13.0)	0.9

Rural-urban disparities

Of the 1,138 patients, 55 patients (5%) resided in a rural area and 1,083 patients (95%) resided in an urban area. In the bivariate screen, age, BMI, EDS grade normal, and EDS grade mild met the criteria for inclusion in our multivariable logistic regression model (Table 1). Multivariable logistic regression analysis showed that older age (OR 1.026, 95% CI 1.000-1.053), lower BMI (OR 0.933, 95% CI 0.878-0.991), and EDS grade normal (OR 5.493, 95% CI 1.645-18.340) were independently associated with rural residence (Table 2).

**Table 2:** Multivariable logistic regression analysis for variables associated with rural residence in patients undergoing carpal tunnel release. ^†^Reference for age and BMI were minus 1 unit. BMI, body mass index; EDS, electrodiagnostic study.

	Multivariable logistic regression		
	Odds ratio	95% confidence interval	p-Value
Age^†^	1.026	(1.000-1.053)	<0.05
Body mass index^†^	0.933	(0.878-0.991)	<0.05
EDS grade normal	5.493	(1.645-18.340)	<0.05
EDS grade mild	0.441	(0.130-1.503)	0.2

## Discussion

There has been growing attention to the need to reduce rural-urban gaps in healthcare outcomes. Several recent studies have demonstrated lower quality of care and higher mortality rates in cardiac disease and stroke among rural patients compared with urban patients [8-10]. No study has previously assessed rural-urban differences in the population of patients undergoing hand surgery in general or CTR in particular. The objectives of this study were to determine whether rural and urban patients who present for CTR are demographically similar, whether they undergo confirmatory EDS testing at the same rate, and whether they present in similar electrodiagnostic stages of disease. We have demonstrated that rural patients who present for CTR are older, have lower BMI, and have a higher rate of EDS grade normal disease.

We have found no difference in the utilization of confirmatory EDS testing between rural and urban patients undergoing CTR at an urban medical center. This lack of disparity is encouraging, because EDS testing is a major preoperative cost driver [17] and a frequent reason for delay to surgical treatment [18]. Our study period was approximately one decade ago, but appropriately timed, because the most up-to-date RUCA codes are based on the 2010 decennial US census data [13]. However, in the past decade, our understanding of the diagnostic standard for CTS has evolved [19-21]. While EDS was traditionally used as a reference standard, it carries measurable false-positive and false-negative rates [2,19], and the American Association of Orthopaedic Surgeons (AAOS) changed its Clinical Practice Guidelines (CPG) on CTS in 2016 to reflect that “the most appropriate setting for electrodiagnostic testing is where there is uncertainty about the clinical diagnosis” [22]. Moreover, some authors advocate for diagnostic aids such as the CTS-6 or ultrasound as alternative standards [2-4]. Therefore, continued investigation on rural-urban differences in confirmatory testing for CTS and timing of surgery is needed to ensure equitable access to care.

We have demonstrated that rural patients undergoing CTR at an urban medical center are older and have lower BMI than their urban counterparts. There are multiple potential explanations for these demographic differences. These differences may simply reflect population-level differences between rural and urban inhabitants, as rural inhabitants are known to be older in general. Data from the US Department of Agriculture shows that 19% of the rural US population is 65 years of age or older compared with 15% of the urban US population [23]. Alternatively, these demographic differences may reflect differing occupations among rural and urban patients with varying incidences of CTS [24-26].

Rural patients were not more likely to present in the advanced electrodiagnostic stages of disease, which is positive and encouraging. This may imply that rural patients have reasonable access to care for CTS in our center for this elective ambulatory surgical treatment that requires low resources. Conversely, we have demonstrated that rural patients undergoing CTR at an urban medical center are more likely to have EDS grade normal disease. Of the entire cohort of 1,138 patients in this study, the rate of EDS grade normal disease was 3%; however, among the rural cohort, one in eight patients had EDS grade normal disease. Possible reasons for this difference include patient preferences, ease of access, and availability of transportation. By definition, rural patients reside in geographically diffuse locations with longer commuting distances. One possible explanation for the higher proportion of EDS-negative CTS in the rural population is that CTS symptoms manifest in these patients during driving while commuting longer distances. A second possible explanation is that surgeons and/or patients are more willing to undergo a more invasive and more definitive treatment (i.e., surgery) when the barrier to treatment (i.e., commute) is higher. The high false-negative rate of EDS for CTS in the rural population calls into question its utility as a confirmatory test in this population, and it is possible that CTS-6 or ultrasound may be a more effective confirmatory test in the rural population. Mackenzie et al. performed a retrospective study of 637 patients who underwent CTR, in which 19 patients (3%) had EDS grade normal disease. When comparing preoperative and one-year postoperative QuickDASH functional scores, Mackenzie et al. showed that while the EDS grade normal group improved at one year, they had a significantly smaller mean improvement in QuickDASH scores compared to the group with preoperative EDS abnormalities [27]. Future studies may assess for outcome disparities of CTR between rural and urban patients.

Our study has several limitations. A primary weakness of our study is ascertainment bias. Our study was performed at a single metropolitan tertiary care referral center, which itself is in an urban setting. The rural cohort comprised a small percentage of our total patient population compared to the national average (5% versus 15%) and represents those rural patients who sought care at an urban hospital. Our findings may be biased if the cohort that presented to our tertiary center is not representative of the overall rural population within our region. Second, our study focused only on surgically treated CTS patients. It is possible, for instance, that rural and urban patients have similar rates of EDS grade normal CTS, but urban patients chose nonoperative treatment more frequently. This would not have been captured by our study methodology. Third, rurality is a complex social construct, and while the use of RUCA codes for rural-urban classification is well established, there is a known discordance between RUCA designation and rural-urban self-identification [28]. In this study, we only assessed rurality using RUCA codes, but patient self-perceived rurality of the community they live in may have effects on their health behaviors and choices. Finally, our study did not control for other relevant socioeconomic factors such as income, education, and trust in the medical system, which may be confounding factors.

## Conclusions

We have performed a single-center retrospective cohort study to assess for rural-urban disparities in the surgical treatment of CTS. We have found no disparity in the utilization of confirmatory EDS testing between rural and urban patients undergoing CTR at an urban medical center. Patients undergoing CTR at an urban medical center who reside in rural areas are significantly older with lower BMI. Moreover, rural patients undergoing CTR at an urban medical center are more likely to have EDS grade normal disease, which calls into question the effectiveness of EDS as a confirmatory test in this population. There is an overall paucity of literature on CTR outcomes in a rural population, and further studies on this topic are needed to determine rural-urban inequities in care.
